# Supplementary material for: Modeling SARS-CoV-2 propagation using rat coronavirus-associated shedding and transmission
Source: PLoS One. 2021 Nov 23;16(11):e0260038. doi: 10.1371/journal.pone.0260038 (PMC8610237; doi:10.1371/journal.pone.0260038)
Supplement: S1 Fig — (DOCX) [file pone.0260038.s001.docx]

**
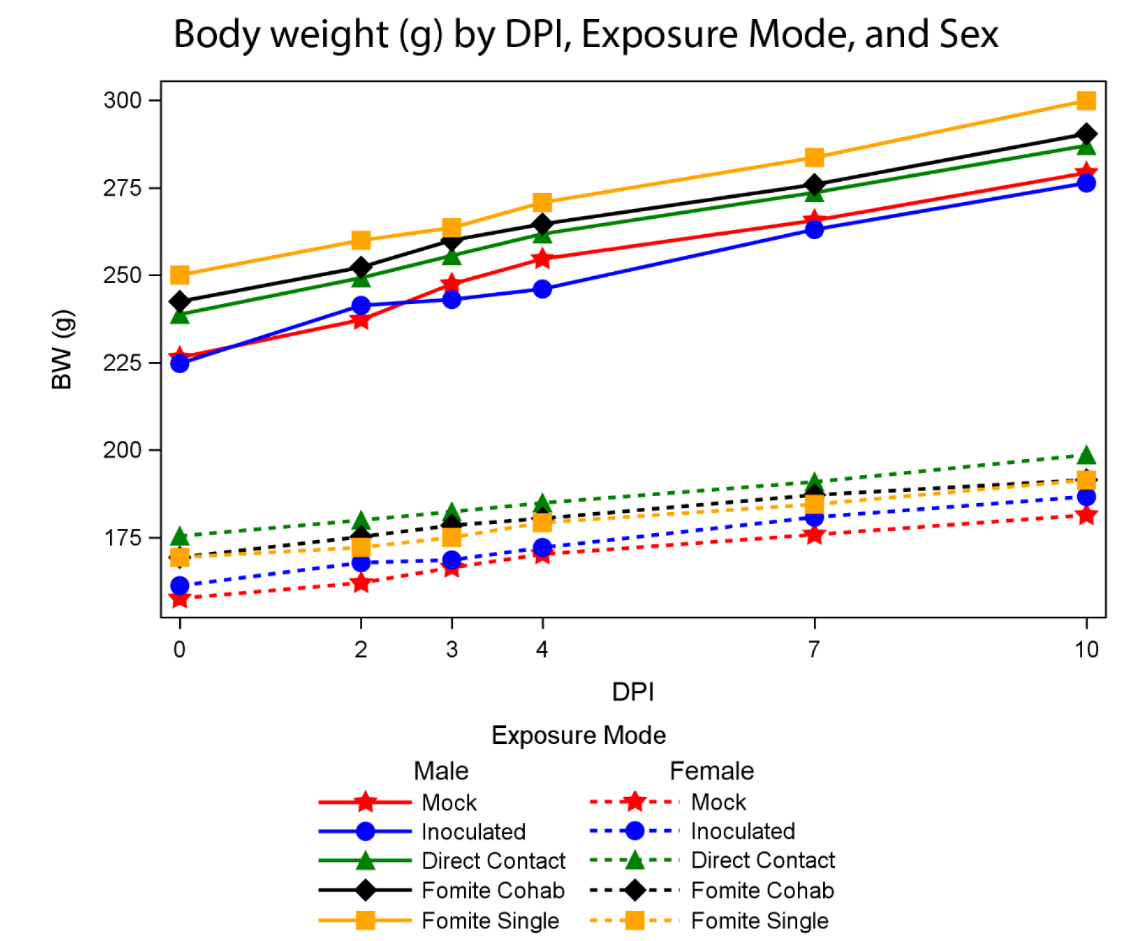
**

**S1 Figure.** Body weight by exposure mode after initial infection with SDAV

Compared to mock-inoculated animals, SDAV-inoculated rats experienced declines in weight gain at days 2-4 post infection. Weight gain slowed during days 2 – 4 regardless of exposure mode, however, only index rats grew significantly less quickly than mock inoculated control animals (p<.001). Male and female rats experienced no significant difference in growth rates.

Body weight was modeled with fixed effects of sex, exposure mode, time, and a mode by time interaction using mixed repeated measures linear models with an autoregressive covariance structure (AR(1)) accounting for correlations of repeated measurement within animal. A second model on the subset of index exposed rats was run to test whether the pronounced slowing of growth differed by sex. But the interaction was not significant, indicating similar slowed growth in male and female rats. Solid lines depict male rats and dashed lines depict female rats.
