# Supplementary material for: Modeling SARS-CoV-2 propagation using rat coronavirus-associated shedding and transmission
Source: PLoS One. 2021 Nov 23;16(11):e0260038. doi: 10.1371/journal.pone.0260038 (PMC8610237; doi:10.1371/journal.pone.0260038)
Supplement: S2 Fig — (DOCX) [file pone.0260038.s002.docx]

**
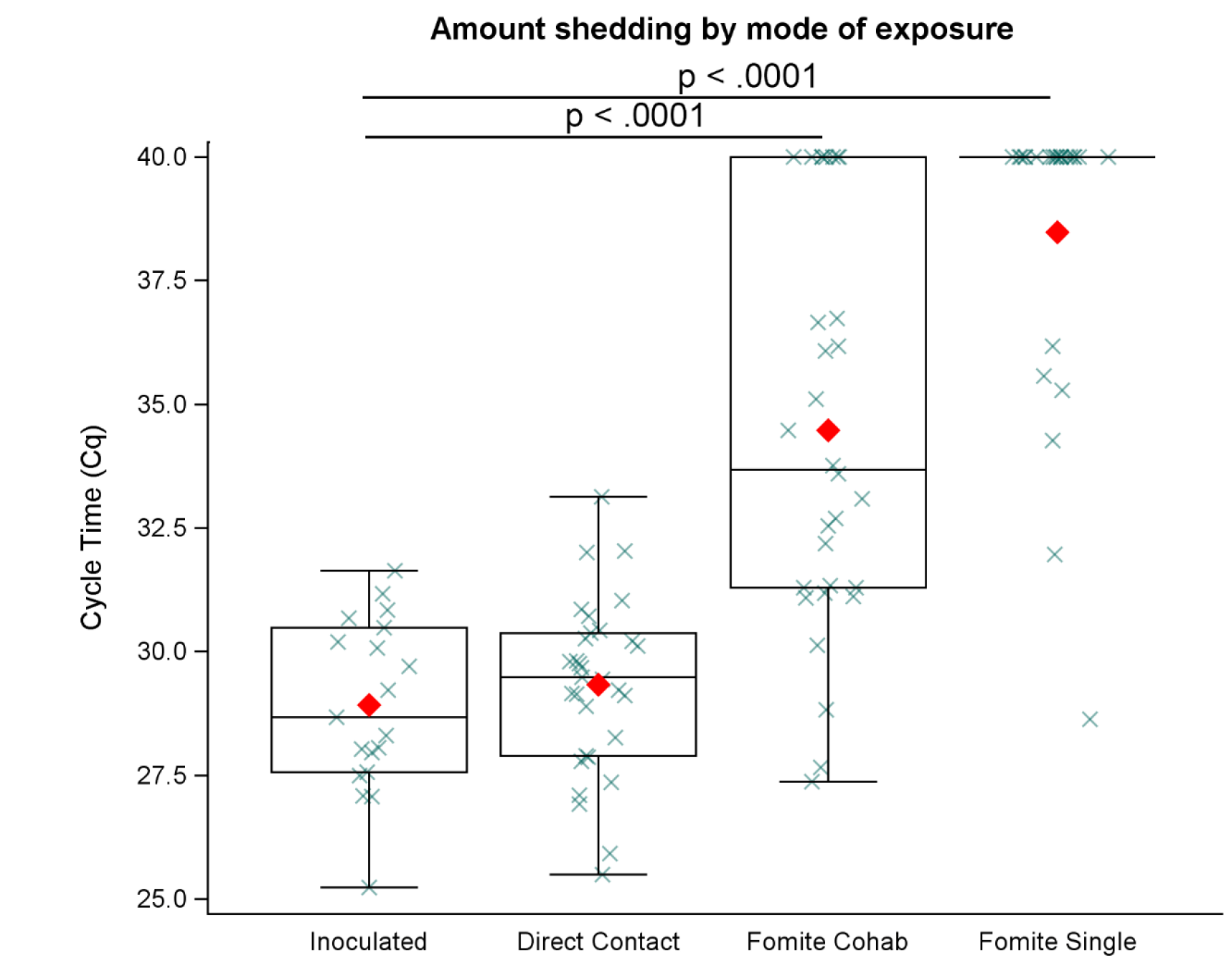
**

**S2 Figure.** Amount of viral shedding following initial SDAV exposure by exposure route.

Route of exposure significantly influenced amount of viral shedding (p<0.0001). Extent of viral

shedding did not differ significantly between index and direct contact groups. Using the

inoculated group as a reference, viral shedding following fomite exposure (both cohabitation and

single groups) was significantly lower (p<.0001). Linear regression modeled the lowest observed

PCR as function of exposure mode, with planned contrasts between exposure modes tested with

t-tests. Red diamonds indicate group means. Individual rat data are depicted with green x-marks.
