# Supplementary material for: Modeling SARS-CoV-2 propagation using rat coronavirus-associated shedding and transmission
Source: PLoS One. 2021 Nov 23;16(11):e0260038. doi: 10.1371/journal.pone.0260038 (PMC8610237; doi:10.1371/journal.pone.0260038)
Supplement: S3 Fig — (DOCX) [file pone.0260038.s003.docx]

**
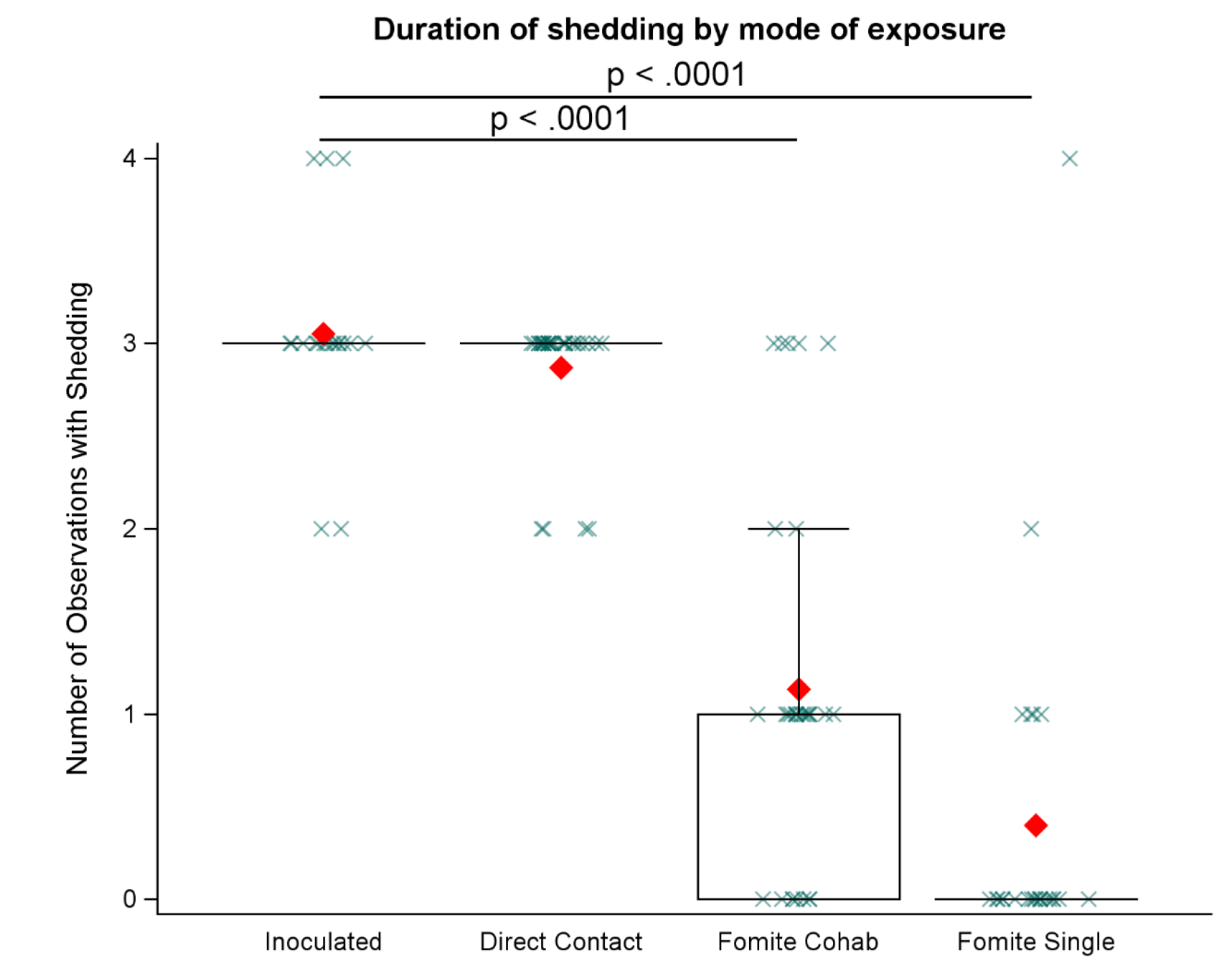
**

**S3 Figure.** Duration of viral shedding following initial SDAV exposure by exposure route.

The count of observations with shedding was modeled with a Poisson linear regression with a

log link as a function of exposure mode. Overall, exposure mode was significant (p<.0001).

Inoculated and direct exposure groups shed virus for approximately twice as long (average 3.1

and 2.9 days respectively) as fomite exposed animals (average 1.5 days, significant at p<0.0001

for fomite-cohabitation group compared to inoculation group). Red diamonds indicate group

means. Individual rat data are depicted with green x-marks.
