# Supplementary material for: Modeling SARS-CoV-2 propagation using rat coronavirus-associated shedding and transmission
Source: PLoS One. 2021 Nov 23;16(11):e0260038. doi: 10.1371/journal.pone.0260038 (PMC8610237; doi:10.1371/journal.pone.0260038)
Supplement: S4 Fig — (DOCX) [file pone.0260038.s004.docx]

**
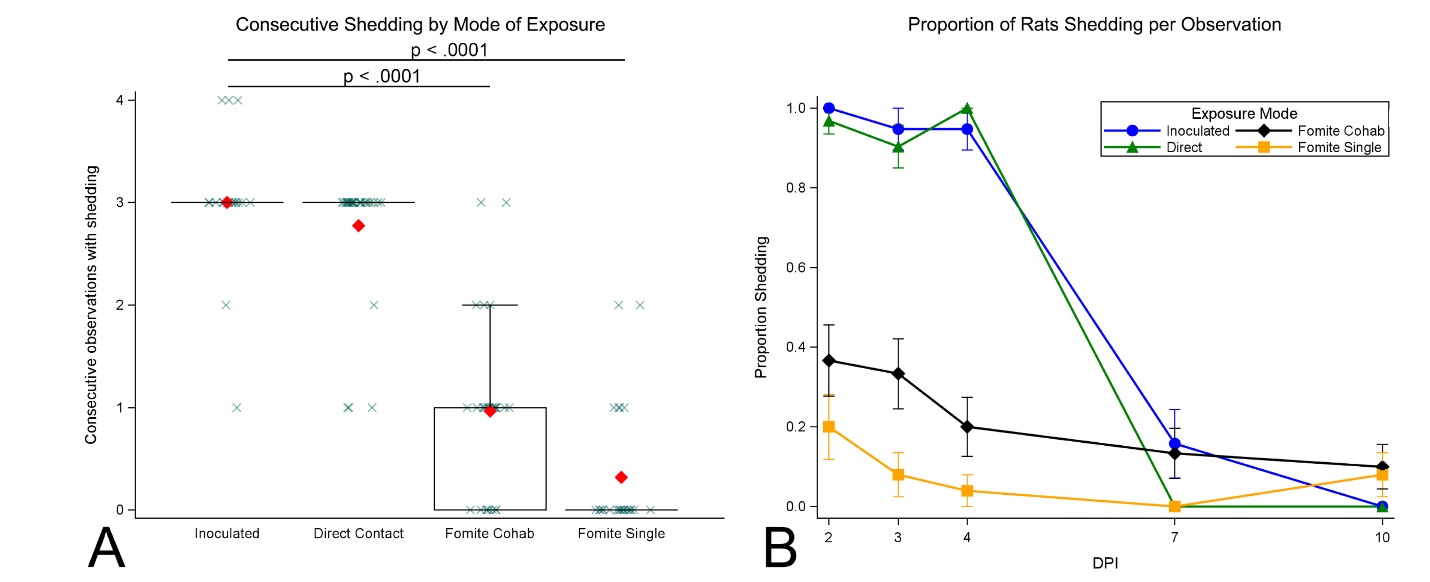
**

**S4 Figure:** Proportion of rats shedding SDAV per observation time by exposure route

Inoculated and direct contact animals shed virus from days 2-4 days post inoculation, with few

animals shedding at day 7 and none at day 10. Fomite exposed animals shed intermittently,

however shedding more commonly persisted to 10 dpi.

Panel A: The count of consecutive observations with shedding was modeled with a Poisson

linear regression with a log link as a function of exposure mode. Consistency of shedding was

significantly affected by exposure mode (p < .0001), with inoculated and direct exposure groups

shedding with significantly greater consistency than fomite-cohabitation (p<.0001) and fomite

single groups (p<.0001). Red diamonds indicate group means. Individual rat data are depicted

with green x-marks.

Panel B depicts the proportion of rats shedding within each exposure mode for each observation

period.
