# Supplementary material for: Modeling SARS-CoV-2 propagation using rat coronavirus-associated shedding and transmission
Source: PLoS One. 2021 Nov 23;16(11):e0260038. doi: 10.1371/journal.pone.0260038 (PMC8610237; doi:10.1371/journal.pone.0260038)
Supplement: S5 Fig — (DOCX) [file pone.0260038.s005.docx]

**
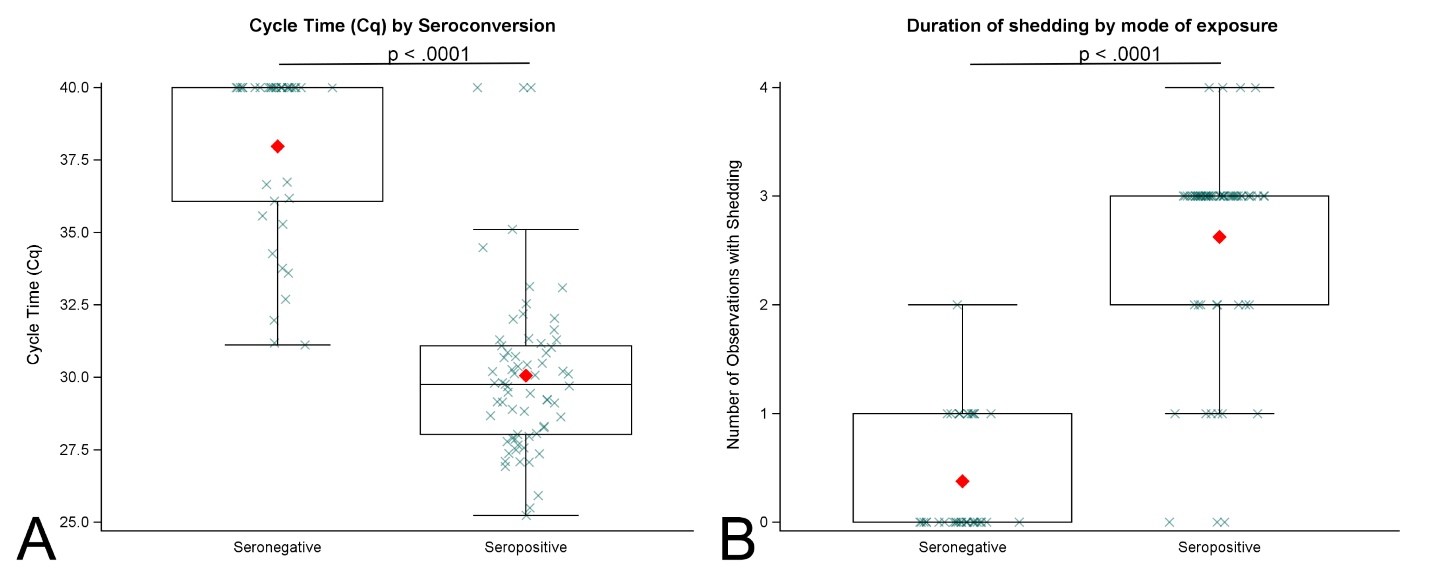
**

**S5 Figure:** Relationship between amount of viral shedding and seroconversion.

Seroconversion was significantly associated with greater amounts (A; p<.0001) and duration (B;

p<.0001) of viral shedding. Sex did not significantly affect viral shedding amount, duration or

seroconversion. Differences between shedding as a function of seroconversion was assessed

using a t-test on the observed lowest cycle times (Cq). Differences between number of

observations with shedding as a function of seroconversion was assessed using a non-parametric

test of medians. Red diamonds indicate group means. Individual rat data are depicted with green

x-marks.

Boxplot of lowest observed Cq by seroconversion status
