# Supplementary material for: Modeling SARS-CoV-2 propagation using rat coronavirus-associated shedding and transmission
Source: PLoS One. 2021 Nov 23;16(11):e0260038. doi: 10.1371/journal.pone.0260038 (PMC8610237; doi:10.1371/journal.pone.0260038)
Supplement: S6 Fig — (DOCX) [file pone.0260038.s006.docx]

**
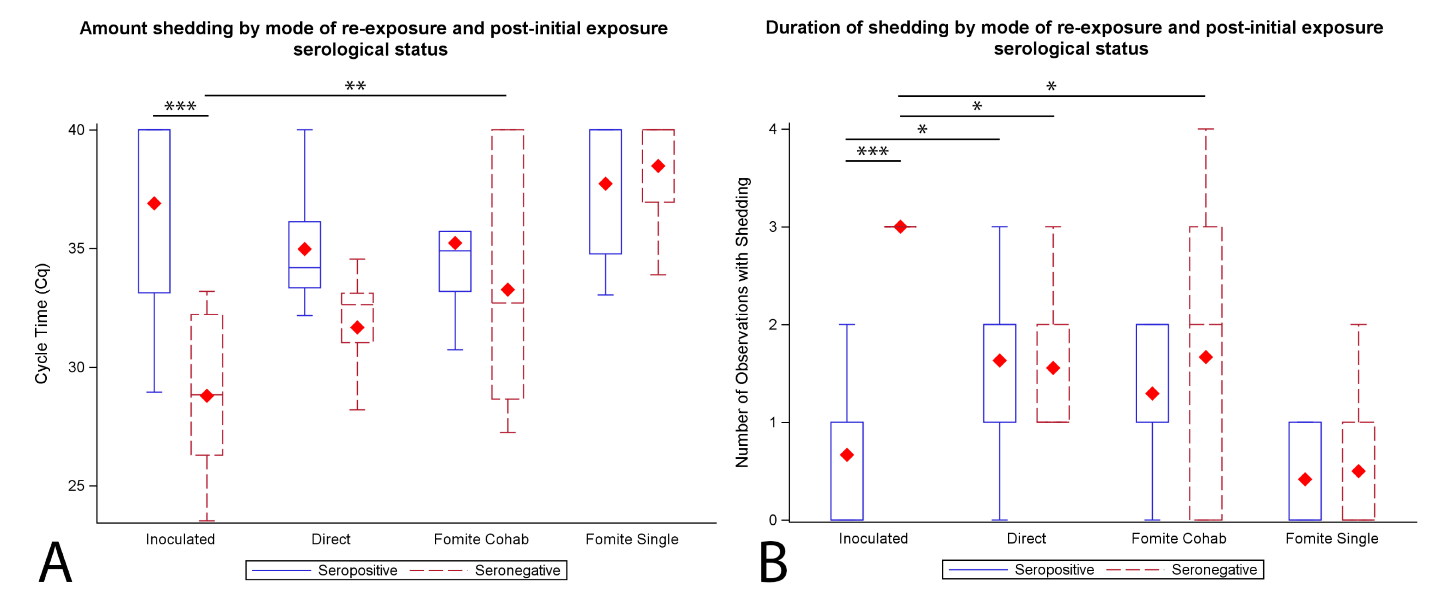
**

**S6 Figure:** Viral shedding on SDAV re-exposure.

1. Amount of viral shedding (expressed as lowest observed Cq) by serologic status and exposure mode. Amount of viral shedding was significantly influenced by both exposure mode (p< .01) and serologic status (p<.001) using linear regression. Previously SDAV-exposed seropositive rats shed less virus (p <.001) compared to previously mock-inoculated seronegative rats. Among seronegative rats, exposure mode significantly affected amount (p < .001) of viral shedding. Compared to seronegative SDAV-inoculated rats, direct contact (p=.06) and fomite cohabitation animals(p<.01) shed less virus. Among seropositive rats, direct and fomite cohabitating rats experienced higher levels of shedding than the SDAV-inoculated seropositive rats, implying greater protection against shedding in the latter. Despite this observation, the amount of viral shedding was not significantly associated with exposure mode (p=.09). Red diamonds indicate group means. Contrasts are marked by asterisks; * p<.05, ** p<.01, *** p<.001.

Boxplot of lowest observed Cq over the entire 10-day observation period by exposure mode

1. Duration of viral shedding (observed shedding events) by serologic status and exposure mode. Duration of shedding was significantly influenced by both exposure mode (p< .05) and serologic status (p<.001) using Poisson regression. Previously SDAV-exposed seropositive rats shed virus for a shorter period (p <.001) compared to previously mock-inoculated seronegative rats when re-inoculated with the same dose of SDAV. Among seronegative rats, exposure mode significantly affected duration (p < .01) of viral shedding. Compared to seronegative SDAV-inoculated rats, direct contact rats shed virus for a fewer observations (p<0.05), as did fomite cohabitation animals (p<0.05). Among seropositive rats, overall exposure mode was significantly (p<.01) associated with duration of shedding. SDAV-inoculated rats generally shed for fewer observations, and significantly fewer observations the direct contact group (p<.05). Red diamonds indicate group means. Contrasts are marked by asterisks; * p<.05, ** p<.01, *** p<.001.
